# Dietary Management of IgE-Mediated and Non-IgE-Mediated Food Allergies: A Systematic Review of Current Evidence and Clinical Implications

**DOI:** 10.3390/nu18193180

**Published:** 2026-09-26

**Authors:** Alejandro Raúl Gratacós Gómez, Esther Iniesta Tejera, Laura Bas Bellver, Sergio Serrano de Pablo, Miriam Clar Castelló, Elisa Gomez Torrijos

**Affiliations:** 1 Servicio de Endocrinologia y Nutrición del Hospital Universitario de Toledo, 45071 Toledo, Spain; 2 Servicio de Alergología del Hospital General Universitario de Ciudad Real, 13005 Ciudad Real, Spain

**Keywords:** food allergy, dietary management, elimination diet, oral immunotherapy, nutrition, IgE-mediated food allergy, non-IgE-mediated food allergy, systematic review

## Abstract

Background: Food allergy is an increasing global health problem associated with substantial clinical, nutritional, and psychosocial burden. Methods: This systematic review was conducted according to the PRISMA 2020 Statement. PubMed/MEDLINE and Scopus were systematically searched for studies published between January 2016 and December 2025. A total of 218 records were identified, of which 180 were screened after duplicate removal; 14 studies met the predefined eligibility criteria and were included in the qualitative synthesis. Methodological quality was assessed using validated appraisal tools according to study design, and findings were synthesized narratively following the SWIM guideline. Results: Avoidance of confirmed culprit foods remains a central component of food allergy management, although prolonged or unnecessarily restrictive elimination may adversely affect dietary diversity, nutritional adequacy, growth, and quality of life, particularly in children and patients with multiple food allergies. Randomized controlled trials and systematic reviews support early allergen introduction for prevention of IgE-mediated food allergies and oral immunotherapy as an active treatment option in selected patients with IgE-mediated food allergies, although treatment-related adverse events remain an important consideration. In contrast, evidence supporting several dietary management strategies for non-IgE-mediated food allergies is more frequently derived from observational studies and evidence-based recommendations, highlighting important gaps in the current evidence base. Conclusions: Current evidence supports individualized dietary management based on regular reassessment, nutritional monitoring, supervised food reintroduction when clinically appropriate, and multidisciplinary care. The evidence supporting different dietary strategies remains heterogeneous, particularly for non-IgE-mediated food allergies, emphasizing the need for further high-quality research.

## 1. Introduction

Food allergies are a major global public health concern, with increasing prevalence in both pediatric and adult populations. Although prevalence estimates vary according to diagnostic criteria, current evidence suggests that food allergy affects approximately 6–10% of children and 2–5% of adults, with the highest burden reported in industrialized countries [1,2,3,4,5,6]. Its increasing prevalence is likely related to complex interactions between genetic susceptibility and environmental factors, including dietary changes, reduced microbial exposure during early life, alterations in the intestinal microbiota, and urbanization [1,2,3].

Food allergy also represents a substantial clinical and healthcare burden, requiring specialist consultations, diagnostic investigations, and, in some cases, emergency care following accidental allergen exposure or anaphylaxis. Its heterogeneous immunopathogenesis, including IgE-mediated, non-IgE-mediated, and mixed mechanisms, further complicates diagnosis and long-term management [6,7,8,9,10]. The main clinical and diagnostic characteristics distinguishing IgE-mediated from non-IgE-mediated food allergies are summarized in Table 1. Non-IgE-mediated food allergies encompass a heterogeneous group of disorders that can affect patients across different age groups, although several of these conditions, particularly food protein-induced gastrointestinal disorders, are most recognized during infancy and early childhood [9,10]. Dietary management remains central to the management of both IgE-mediated and non-IgE-mediated food allergies in both children and adults. Although elimination of culprit foods can prevent allergic reactions, prolonged or unnecessarily restrictive diets may compromise nutritional adequacy, dietary diversity, growth, and eating behaviors, particularly in children and when multiple nutritionally important foods are excluded [3,11,12]. Dietary restrictions may also negatively affect social activities and health-related quality of life, highlighting the need for individualized nutritional assessment and ongoing dietetic support [13,14,15,16].

Over the last decade, food allergy management has undergone a substantial paradigm shift. Although allergen avoidance remains fundamental, increasing evidence supports active prevention and strategies aimed at promoting immune tolerance. Landmark trials have demonstrated that early introduction of allergenic foods during infancy can reduce the risk of developing food allergy, leading to changes in international prevention guidelines [17,18,19]. Oral immunotherapy has also emerged as a treatment option for selected patients with IgE-mediated food allergies, although safety, treatment burden, and long-term efficacy remain important considerations [19]. In non-IgE-mediated food allergies, dietary management is mainly based on individualized food avoidance, nutritional support, and supervised food reintroduction when appropriate [20].

Despite these advances, evidence on dietary management remains fragmented across food allergy phenotypes, age groups, interventions, and outcomes. Previous systematic reviews and guidelines have addressed specific aspects of food allergy management, including dietary elimination, early allergen introduction, and oral immunotherapy [17,18,19,20]. However, an integrated synthesis of dietary management across both IgE-mediated and non-IgE-mediated food allergies remains limited. The publication of major randomized controlled trials and updated clinical practice guidelines in recent years further supports the need for an updated synthesis of the available evidence.

The aim of this systematic review was to synthesize current evidence on dietary management strategies for IgE-mediated and non-IgE-mediated food allergies, including allergen elimination, nutritional management, food reintroduction, oral immunotherapy, early allergen introduction, and multidisciplinary nutritional care. We also evaluated their clinical and nutritional implications, including symptom control, tolerance or desensitization, nutritional status and growth, adverse events, dietary diversity, and quality of life.

The review question was structured according to the PICO framework. The population comprised patients with IgE-mediated or non-IgE-mediated food allergies. Interventions included dietary elimination; oral immunotherapy; early allergen introduction; supervised or structured food reintroduction, including allergen ladders; and multidisciplinary nutritional management. Comparators included usual care, alternative dietary strategies, or no intervention, when applicable. Outcomes included clinical outcomes, nutritional status and growth, development of tolerance, treatment-related adverse events, and quality of life.

## 2. Methods

### 2.1. Study Design

This systematic review was conducted and reported in accordance with the Preferred Reporting Items for Systematic Reviews and Meta-Analyses (PRISMA 2020, PROSPERO 24-25) Statement [21]. The PRISMA 2020 Explanation and Elaboration Document [22] and the Cochrane Handbook for Systematic Reviews of Interventions (Version 6.4) [23] were used to guide the design, conduct, and reporting of this review.

#### Protocol Registration

This systematic review was not prospectively registered in the International Prospective Register of Systematic Reviews (Prospero). The absence of prospective registration is acknowledged as a methodological limitation of this review.

### 2.2. Literature Search Strategy

A comprehensive literature search was performed in Pubmed and Scopus, selected because of their extensive coverage of the biomedical and multidisciplinary scientific literature. Studies published between January 2016 and December 2025 were considered eligible to provide an updated synthesis of the evidence on dietary management strategies for IgE-mediated and non-IgE-mediated food allergies. The electronic searches were performed on 10 January 2026.

The search strategy was developed using a combination of Medical Subject Headings (MeSH) and free-text terms. The principal search concepts included “Food Hypersensitivity,” “Food Allergy,” “IgE-mediated food allergy,” “Non-IgE-mediated food allergy,” “Elimination diet,” “Dietary management,” “Food Protein-Induced Enterocolitis Syndrome (FPIES),” “Eosinophilic Esophagitis,” “Nutritional management,” “Quality of life,” and “Food allergy quality of life.” Boolean operators (AND, OR) were used to combine search terms, with search strategies adapted to the indexing system of each database. A single comprehensive search strategy was used to identify evidence across the predefined dietary management domains, rather than conducting separate independent searches for each intervention category. These terms represent the principal concepts of the search strategy; the complete database-specific search strategies, including additional synonyms and related terms, are provided in Supplementary File S1.

The search strategy was developed according to current recommendations for comprehensive and reproducible literature searches [24,25]. The study selection process is summarized in the PRISMA 2020 flow diagram (Figure 1), and the completed PRISMA 2020 Checklist is provided as Supplementary File S2.

### 2.3. Eligibility Criteria

Inclusion criteria: Studies were eligible if they fulfilled all of the following criteria: (I) included human participants with IgE-mediated and/or non-IgE-mediated food allergies; (II) evaluated a dietary management strategy, including allergen elimination, individualized nutritional management, dietary counseling, oral immunotherapy, supervised food reintroduction, structured food reintroduction approaches (including food or allergen ladders, or other structured dietary approaches); (III) reported at least one clinical, nutritional, growth, food tolerance, adverse-event, dietary diversity, or quality-of-life outcome; (IV) were published in English or Spanish; and (V) were published between January 2016 and December 2025.

Randomized controlled trials, non-randomized intervention studies, prospective or retrospective observational studies, systematic reviews, meta-analyses, evidence-based clinical practice guidelines, and international position papers were eligible for inclusion.

Studies were excluded if they met any of the following criteria: (I) animal or in vitro studies; (II) conference abstracts without full-text availability; (III) editorials, letters to the editor, narrative reviews, or expert opinions without original data; (IV) duplicate publications; (V) studies that did not specifically address dietary or nutritional management of food allergy; or (VI) articles published outside the predefined study period. Background and contextual references used in the Introduction or Discussion were retained in the reference list, but were not counted as included studies unless they fulfilled all predefined eligibility criteria.

### 2.4. Study Selection

Study selection was performed independently by two reviewers in two consecutive phases. During the first phase, titles and abstracts retrieved from the electronic searches were independently screened according to the predefined eligibility criteria. In the second phase, the full texts of potentially eligible studies were independently assessed by the same reviewers to determine their final eligibility for inclusion. Any disagreements were resolved through discussion until consensus was reached. The study selection process is summarized in the PRISMA 2020 flow diagram (Figure 1).

### 2.5. Data Extraction

Data extraction was independently performed by two reviewers using a standardized data extraction form specifically developed for this review. The following variables were extracted from each eligible study: first author, year of publication, country, study design, population characteristics, type of food allergy (IgE-mediated or non-IgE-mediated), dietary intervention or elimination strategy, specific food allergen(s) involved, duration of dietary restriction, food reintroduction protocols, clinical outcomes, nutritional outcomes, growth and anthropometric outcomes, dietary diversity, adverse events, quality-of-life outcomes, and the main conclusions. For the purposes of evidence synthesis, primary outcomes were defined as clinical outcomes related to dietary management, including symptom control, successful food reintroduction, development of tolerance or desensitization, and changes in reaction thresholds, as applicable. Secondary outcomes included nutritional status, growth and anthropometric measures, dietary diversity, treatment-related adverse events, and quality of life. Any discrepancies identified during data extraction were resolved through discussion until consensus was achieved. Extracted data were cross-checked for accuracy before synthesis.

### 2.6. Quality Assessment of Included Studies

Randomized controlled trials were evaluated using the Cochrane Risk of Bias 2 (ROB 2) tool; observational cohort and case–control studies were assessed using the Newcastle–Ottawa Scale (NOS); and systematic reviews were appraised using the AMSTAR 2 (A Measurement Tool to Assess Systematic Reviews 2) instrument. International clinical practice guidelines were assessed using the Appraisal of Guidelines for Research and Evaluation II (AGREE II) Framework. Position papers were critically appraised according to their methodological rigor, transparency of development, supporting evidence, and consistency with current international recommendations. Given the broad scope of this review and the inclusion of multiple study designs, the quality assessment was used to support the interpretation of evidence, rather than to exclude studies from qualitative synthesis. Quality assessment was independently performed by two reviewers, with disagreements resolved through discussion until consensus was reached.

Importantly, the different evidence sources were not considered equivalent in evidentiary strength; randomized controlled trials and high-quality systematic reviews were prioritized when available, whereas clinical practice guidelines and position papers were used primarily to contextualize recommendations and current clinical practice. The conclusions and recommendations of secondary evidence sources were interpreted in conjunction with their methodological quality and the supporting evidence reported by the original authors.

### 2.7. Data Synthesis

Because of the substantial clinical and methodological heterogeneity among the included studies regarding study design, patient populations, dietary interventions, and outcome measures, a quantitative meta-analysis was not considered appropriate. Therefore, findings were synthesized using a structured narrative synthesis, following the recommendations of the SWIM (Synthesis Without Meta-Analysis) reporting guideline for systematic reviews without meta-analysis [26]. The interpretation of findings considered study design, methodological quality, population characteristics, and the consistency of results across evidence sources. The synthesis was organized according to the type of food allergy (IgE-mediated and non-IgE-mediated), dietary management strategy, clinical outcomes, nutritional outcomes, food reintroduction and tolerance, treatment-related adverse events, and quality-of-life outcomes.

### 2.8. Ethical Considerations

As this study consisted exclusively of a systematic review of the previously published literature, approval from an institutional ethics committee and informed consent were not required.

## 3. Results

### 3.1. Study Selection

The literature search identified a total of 218 records, including 163 retrieved from Pubmed and 55 from Scopus. After removal of 38 duplicate records, 180 articles were screened by title and abstract according to the predefined eligibility criteria. Of these, 135 records were excluded. Full-text assessment was performed for 45 articles, of which 31 were excluded for not meeting the inclusion criteria. Finally, 14 studies were included in qualitative synthesis. The study selection process is summarized in the PRISMA 2020 flow diagram (Figure 1).

### 3.2. Characteristics of Included Studies

The evidence base comprised randomized controlled trials, observational studies, systematic reviews and meta-analyses, international clinical guidelines, and evidence-based position papers addressing different aspects of dietary management in food allergies. Considerable heterogeneity was observed in study design, population, intervention, and reported outcomes. The evidence base included a substantial proportion of studies involving pediatric populations, particularly infants, children, and adolescents, while evidence specifically addressing adults was more limited. Accordingly, the evidence was synthesized according to predefined clinical domains, including elimination and dietary restriction, nutritional consequences and dietary counseling, oral immunotherapy, structured food reintroduction, and preventive dietary strategies, following SWIM guidance [26]. Table 2 presents the characteristics of all 14 studies and evidence sources included in qualitative synthesis.

### 3.3. Methodological Quality of Included Studies

The methodological quality of the included evidence varied according to study design and the assessment approach applied. Systematic reviews and meta-analyses included in the methodological appraisal generally showed high methodological quality according to AMSTAR 2. Randomized controlled trials showed low risk of bias or some concerns according to ROB 2, depending on the study. International clinical practice guidelines generally showed high or moderate-to-high methodological quality according to the AGREE II Framework or structured guideline appraisal. Position papers were critically appraised considering their methodological rigor, transparency of development, supporting evidence, and consistency with current international recommendations.

### 3.4. Elimination Diets and Allergen Avoidance

Elimination diets were the most consistently represented dietary management strategy across the included evidence. Their primary role was to prevent clinical reactions by excluding confirmed culprit foods, particularly in patients with IgE-mediated food allergies, while in non-IgE-mediated disorders, elimination diets were used as part of the diagnostic and therapeutic approaches [6,20,36].

The extent and duration of dietary restriction varied substantially across the included evidence. Current evidence supports maintaining tolerated foods in the diet and providing individualized dietary advice to minimize unnecessary restriction and preserve dietary diversity [20,27].

Prolonged or multiple-food elimination diets may reduce dietary diversity and increase the risk of inadequate nutrient intake, particularly when nutritionally important foods are excluded without appropriate substitution. The specific nutritional risk depends on the food excluded; for example, cow’s milk elimination may compromise calcium, vitamin D, and protein intake [11,27].

The available evidence supports the use of targeted, rather than unnecessarily restrictive, elimination diets, with regular reassessment of the clinical indication for continued exclusion. Where clinically appropriate, supervised food reintroduction may help reassess tolerance and reduce unnecessary long-term dietary restriction [20,27].

### 3.5. Nutritional Consequences of Dietary Restriction

Nutritional consequences were an important outcome across the included evidence, particularly among children undergoing prolonged or multiple-food elimination. Restrictive dietary patterns may reduce dietary diversity and increase the risks of inadequate nutrient intake and impaired growth when multiple foods or nutritionally important foods are excluded without appropriate substitution [3,11,27,37].

Nutritional assessment and anthropometric monitoring were identified as important components of dietary management. Individualized dietary counseling and appropriate nutritional substitution may help maintain nutritional adequacy and reduce unnecessary food avoidance [11,27].

Overall, the available evidence supports individualized nutritional management and regular reassessment of dietary restrictions to minimize nutritional consequences while maintaining adequate control of food allergies [11,20,27].

### 3.6. Oral Immunotherapy and Active Treatment Strategies

Oral immunotherapy (OIT) was the most extensively studied active treatment approach identified in the included evidence. Randomized controlled trials and systematic reviews demonstrated that OIT can increase desensitization rates in patients with IgE-mediated food allergies compared with allergen avoidance or placebo [28,29,30,31,38].

The available evidence also indicated that OIT is associated with treatment-related adverse events, although the frequency and severity varied among studies [28,29,30,31]. These findings highlight the importance of careful patient selection and appropriate clinical supervision during treatment [19,28,29,31,32].

Current EAACI recommendations support allergen-specific immunotherapy as an active treatment option for appropriately selected patients with IgE-mediated food allergies. Peanut OIT is recommended for children and adolescents with peanut allergies to achieve desensitization, while OIT for egg and cow’s milk allergies is suggested in selected children and adolescents [19,32].

Overall, the evidence supports OIT as an effective approach for increasing the threshold of reaction during ongoing treatment, although the evidence for sustained unresponsiveness after treatment discontinuation remains less established [28,29,30,31,32].

### 3.7. Early Allergen Introduction and Prevention

Early introduction of allergenic foods during complementary feeding in infancy was identified as an important preventive strategy for IgE-mediated food allergies. The landmark LEAP trial, published before the predefined inclusion period of this review, provided pivotal background evidence that early introduction of peanut in high-risk infants significantly reduced the subsequent development of peanut allergy [17,39]. Within the predefined inclusion period, the EAT study evaluated the early introduction of multiple allergenic foods and suggested a potential reduction in the development of food allergy, although adherence to the intervention influenced the observed effect [33].

These findings have contributed to a shift in preventive dietary recommendations, with current international guidelines supporting the introduction of allergenic foods during infancy, rather than prolonged avoidance [12,17,19]. The available evidence supports early allergen introduction as a preventive dietary strategy, particularly for peanuts and eggs, although the applicability of these findings to other foods and populations remains less certain.

### 3.8. Nutritional Counseling and Multidisciplinary Care

Individualized nutritional counseling was identified as an important component of dietary management in food allergy. The available evidence emphasized the role of individualized dietary assessment and appropriate nutritional substitution, particularly in patients requiring prolonged or multiple-food elimination diets [3,27].

Dietitian-led dietary management may help reduce unnecessary food avoidance while maintaining nutritional adequacy [20,27]. These interventions are particularly relevant in children, in whom nutritional requirements are high and restrictive diets may have a greater impact on nutrient intake and growth [11].

The evidence also supported a multidisciplinary approach involving allergists, dietitians, and other healthcare professionals when appropriate. Such collaboration may facilitate individualized dietary planning, nutritional monitoring, assessment of food reintroduction, and ongoing reassessment of the need for dietary restriction [20,27].

Overall, the available evidence supports integrating individualized nutritional counseling and multidisciplinary follow-up into comprehensive dietary management, particularly for patients with multiple food allergies or prolonged dietary restrictions [11,20,27]. The principal dietary management strategies and their clinical implications are summarized in Table 3.

### 3.9. Nutritional and Psychosocial Impacts

Food allergies affect not only nutritional status, but also psychosocial well-being and quality of life. Restrictive diets may be associated with anxiety, social limitations, and reduced enjoyment of food, particularly in children and adolescents [27].

The psychosocial burden may be greater in patients with multiple food allergies, in whom dietary restrictions can interfere with everyday activities and social interactions [27]. These findings highlight that the impact of dietary management extends beyond nutritional adequacy and that the psychosocial consequences of food avoidance should also be considered.

## 4. Discussion

The present systematic review indicates that dietary management remains a central component of the treatment of both IgE-mediated and non-IgE-mediated food allergies. However, the current evidence favors an individualized approach to nutritional management, based on regular reassessment, supervised food reintroduction when clinically appropriate, and multidisciplinary care [6,20,27,37]. The evidence also indicates that the benefits of dietary restriction should be balanced against its potential nutritional and psychosocial consequences, particularly when multiple foods are excluded or elimination is prolonged [11,13,14,27]. Overall, the findings support a patient-centered approach, in which dietary interventions are tailored to the type of food allergy, clinical needs, age, and nutritional requirements of each patient [3,27].

A key finding of this systematic review is that elimination diets remain an important component of food allergy management, particularly when avoidance of a clinically relevant allergen is required to prevent reactions or control symptoms [27,36]. However, prolonged or unnecessarily restrictive diets may reduce dietary diversity and compromise nutritional adequacy, particularly in children [11,27,37]. Therefore, dietary restriction should be targeted to clinically relevant foods, while tolerated foods should be maintained whenever possible, and the need for continued elimination should be periodically reassessed [27,37].

The nutritional impact of dietary restriction is particularly relevant in children requiring prolonged elimination diets, as these restrictions may increase the risk of inadequate nutrient intake and impaired growth [11]. This highlights the importance of nutritional assessment and individualized dietary counseling, particularly when elimination diets are prolonged or involve multiple foods [3,27,40]. Dietary management should therefore aim to balance adequate control of allergic disease with preservation of nutritional adequacy and dietary diversity [11,27,37].

Beyond nutritional effects, food allergies impose a substantial psychosocial burden on patients and their families. Anxiety, fear of accidental exposure, and the need for constant vigilance can negatively affect health-related quality of life, particularly in children and adolescents [13,14]. The burden may be greater when dietary restrictions are extensive or involve multiple foods, as food allergies can interfere with everyday activities, social interactions, and food-related experiences [13,14,15]. These findings highlight the importance of considering psychosocial and patient-reported outcomes when assessing the impacts of dietary management, rather than focusing exclusively on nutritional adequacy and clinical symptoms [15,16].

Regarding active treatment strategies, oral immunotherapy (OIT) represents the most extensively studied immunotherapeutic approach for IgE-mediated food allergies. Systematic reviews and randomized controlled trials indicate that OIT can increase the threshold of reaction and induce desensitization in appropriately selected patients [28,29,30,31,38]. As post-search contextual evidence, a 2026 systematic review and meta-analysis reported an overall incidence of confirmed eosinophilic esophagitis (EoE) of 2.31% (95% CI, 1.45–3.36) among patients undergoing food allergen desensitization [41]. Because this publication appeared after the predefined search period, it was not counted among the 14 included studies; it is cited here only to contextualize emerging safety evidence. Persistent gastrointestinal symptoms during OIT should prompt appropriate evaluation for possible EoE. However, OIT is also associated with treatment-related adverse events, highlighting the importance of careful patient selection and specialist supervision [28,31,38]. Current EAACI recommendations support allergen-specific immunotherapy as an active treatment option for appropriately selected patients with IgE-mediated food allergies. Peanut OIT is recommended for children and adolescents with peanut allergies to achieve desensitization, while OIT for egg and cow’s milk allergies is suggested in selected children and adolescents [19,32].

Early introduction of allergenic foods represents an important preventive dietary strategy supported by randomized controlled evidence. The landmark LEAP trial [39], although published before the predefined inclusion period of this review, provided pivotal evidence that early peanut introduction substantially reduced the development of peanut allergy in high-risk infants. Within the predefined study period, the EAT trial evaluated the early introduction of multiple allergenic foods and highlighted the importance of adherence to the intervention [17,33]. These findings have contributed to an evolution in preventive dietary recommendations, with current international guidelines favoring the introduction of allergenic foods during infancy, rather than prolonged allergen avoidance [12,17,18,19]. However, the preventive evidence is largely derived from specific populations and foods, particularly peanut [34,42], and should therefore not be generalized to all food allergies.

An important finding of the present review is that non-IgE-mediated food allergies present distinct diagnostic and nutritional challenges. Diagnosis is often based primarily on the clinical history, response to an elimination diet, and recurrence of symptoms following food reintroduction, as specific validated diagnostic biomarkers are lacking for many non-IgE-mediated food allergic disorders [20,35,43]. This diagnostic uncertainty may contribute to prolonged dietary restriction and highlights the importance of supervised elimination and reintroduction strategies in clinical practice [20,27,35,44].

From a nutritional perspective, the management of non-IgE-mediated food allergies can be particularly challenging because elimination diets may involve nutritionally important foods. Post-search contextual evidence published in 2026 has further highlighted growth outcomes and dietary strategies in these disorders [45]; this publication was not counted among the 14 included studies because it appeared after the predefined search period. Although dietary avoidance is an important therapeutic strategy, prolonged or unnecessarily restrictive elimination may compromise nutrient intake and growth, particularly during infancy and early childhood [11,27]. These concerns underline the need for individualized dietary management, regular nutritional assessment, and appropriate follow-up during elimination and subsequent food reintroduction [20,27,35].

Current recommendations emphasize that elimination diets should be individualized and periodically reassessed, with supervised food reintroduction whenever clinically appropriate [20,27,35]. This approach may help minimize unnecessary dietary restrictions, improve dietary diversity, and support nutritional adequacy, while maintaining appropriate control of symptoms [20,27,37].

Attention should be paid to infants and young children, in whom prolonged exclusion of nutritionally important foods may increase the risk of inadequate nutrient intake during critical periods of growth and development [11,27]. Regular nutritional assessment, anthropometric monitoring, and individualized dietary counseling are therefore important components of dietary management in these patients [11,20,27,37].

The heterogeneous nature of non-IgE-mediated food allergies further supports the need for individualized dietary management. Different disorders may vary substantially in their clinical presentations, disease courses, and nutritional implications; therefore, dietary interventions should be tailored to the specific disease phenotype, rather than applying a uniform elimination strategy [20,35,44]. In addition, prolonged dietary restriction may contribute to feeding difficulties and reduced dietary variety, particularly in infants and young children, reinforcing the importance of regular reassessment and supervised food reintroduction when clinically appropriate [11,20,27].

Another important challenge is the limited availability of validated biomarkers for the diagnosis and monitoring of non-IgE-mediated food allergies. Consequently, clinical assessment and elimination–reintroduction protocols remain central to diagnosis and follow-up, while the lack of reliable objective biomarkers may complicate decisions regarding the duration of dietary elimination and the timing of food reintroduction [20,35,44].

Collectively, these findings reflect an evolution in food allergy management over the past decade, from an emphasis on prolonged allergen avoidance toward more individualized dietary management based on nutritional optimization, supervised food reintroduction, active immunomodulatory therapies, and multidisciplinary care.

One of the main findings of this review is that the quality of the available evidence varies substantially across different dietary management strategies. While recommendations regarding early allergen introduction and oral immunotherapy are supported by high-quality randomized controlled trials and meta-analyses, the dietary management of non-IgE-mediated food allergies continues to rely largely on observational studies, expert consensus, and international position papers. This imbalance highlights important knowledge gaps and reinforces the need for well-designed prospective multicenter studies evaluating nutritional outcomes, food reintroduction strategies, long-term tolerance acquisition, and patient-reported outcomes.

### Strengths and Limitations

This systematic review has several important strengths. It was conducted according to the PRISMA 2020 Statement using a predefined search strategy across two bibliographic databases (Pubmed and Scopus). The search strategy combined controlled vocabulary and free-text terms and followed current recommendations for systematic literature searches, thereby enhancing the transparency and reproducibility of the review process.

In addition, the inclusion of different types of evidence, ranging from randomized controlled trials and observational studies to systematic reviews, meta-analyses, clinical practice guidelines, and position papers, introduces variability in methodological rigor and evidentiary strength. Although the methodological quality of each study type was assessed using appropriate appraisal tools, the qualitative integration of these heterogeneous sources may limit direct comparability and require cautious interpretation of the overall conclusions. Importantly, the different evidence sources were not considered equivalent in evidentiary strength; randomized controlled trials and high-quality systematic reviews were prioritized when available, whereas clinical practice guidelines and position papers were used primarily to contextualize recommendations and current clinical practice.

Although randomized controlled trials provide high internal validity, their findings may not always fully reflect outcomes in routine clinical practice, where patient characteristics, adherence, treatment settings, and follow-up may differ substantially from controlled trial conditions.

Clinical practice guidelines were not considered equivalent to expert opinion; their recommendations were interpreted according to the methodological rigor of their development, the transparency of the consensus process, and the quality of the supporting evidence.

A strength of this review is the integration of evidence across several clinically relevant aspects of dietary management. Unlike previous reviews, focusing on a single aspect of food allergy management, the present study integrates evidence on elimination diets, nutritional consequences, oral immunotherapy, early allergen introduction, quality of life, and multidisciplinary nutritional care in both IgE-mediated and non-IgE-mediated food allergies. The inclusion of randomized controlled trials, observational studies, systematic reviews, meta-analyses, and international evidence-based guidelines provides a broad and clinically relevant overview of current dietary management strategies.

Another strength is the inclusion of recent evidence published during the predefined study period, allowing incorporation of current EAACI recommendations and advances in oral immunotherapy and nutritional management. The methodological quality of the included studies was considered during data synthesis and used to inform the interpretation of the findings according to study design and methodological rigor. Most of the included studies were conducted in pediatric populations, reflecting the greater availability of evidence on food allergy management in children. Although adult populations were also represented, the applicability of some findings to adults may therefore be more limited, which should be considered when interpreting the generalizability of the results across age groups.

Nevertheless, several limitations should be acknowledged. First, a quantitative meta-analysis was not performed because of the substantial clinical and methodological heterogeneity among the included studies. Differences in study populations, diagnostic criteria, food allergens, intervention protocols, outcome measures, and follow-up periods prevented meaningful statistical pooling of the available data. Consequently, the findings are based on a qualitative synthesis of the evidence.

Second, although two bibliographic databases were systematically searched, grey literature, conference proceedings, dissertations, and unpublished studies were not included, which may have introduced publication bias. Furthermore, only articles published in English or Spanish were considered, making language bias possible. In addition, specific search terms for oral immunotherapy and structured food reintroduction approaches, including food or allergen ladders, were not explicitly incorporated into the original electronic search strategy. Although relevant evidence on these interventions was retrieved through the broader dietary management search terms, the absence of intervention-specific search terms may have reduced the sensitivity of the search for these approaches and should be considered a limitation of this review. Third, this review was not prospectively registered in PROSPERO, which represents a limitation because prospective registration enhances protocol transparency and allows verification of prespecified methodological decisions.

A formal assessment of reporting bias was not performed because this review did not include a quantitative meta-analysis, and the included studies were highly heterogeneous in terms of study design, interventions, and reported outcomes. Similarly, the certainty of the evidence was not formally evaluated using the GRADE approach. These limitations should be considered when interpreting the findings and the strength of the conclusions of this review.

Finally, food allergies are a rapidly evolving field. Emerging evidence regarding biologic therapies, precision nutrition, microbiome-targeted interventions, and novel immunotherapeutic strategies may modify current clinical practice. Therefore, periodic updates of the evidence will be important to incorporate future advances and maintain the clinical relevance of dietary management recommendations.

Overall, the findings of this systematic review support the central role of dietary management in food allergy treatment. However, dietary interventions should be individualized according to the underlying immunological mechanism, patient age, nutritional requirements, and disease phenotype. Appropriate dietary counseling provided by experienced healthcare professionals is important to support effective allergen avoidance while maintaining nutritional adequacy, promoting normal growth and development, and preserving quality of life. Furthermore, evidence regarding oral immunotherapy, early allergen introduction, and multidisciplinary care supports the continued development of increasingly individualized and evidence-based dietary management strategies [5,7,13,28].

## 5. Conclusions

This systematic review indicates that dietary management remains a central component of treatment for both IgE-mediated and non-IgE-mediated food allergies. Current evidence favors an individualized approach to nutritional management based on regular reassessment, supervised food reintroduction when clinically appropriate, multidisciplinary care, and preservation of nutritional adequacy.

Although avoidance of confirmed culprit foods is an important component of food allergy management, prolonged or unnecessarily restrictive dietary avoidance may compromise dietary diversity, nutritional adequacy, growth, and quality of life, particularly in children and patients with multiple food allergies. These concerns are particularly relevant in non-IgE-mediated food allergies, where diagnostic uncertainty may contribute to prolonged elimination diets and delayed food reintroduction.

The present review also highlights that the evidence supporting different dietary management strategies is heterogeneous. Oral immunotherapy and early allergen introduction have been evaluated in randomized controlled trials and systematic reviews, whereas evidence for several nutritional approaches in non-IgE-mediated food allergies is more frequently derived from observational studies and evidence-based recommendations. This heterogeneity should be considered when interpreting the findings and highlights the need for further high-quality research.

Overall, dietary management should be viewed not simply as allergen avoidance, but as a dynamic and individualized process aimed at maintaining nutritional adequacy, minimizing unnecessary dietary restriction, facilitating appropriate food reintroduction when clinically indicated, and preserving quality of life. Regular nutritional assessment, individualized dietary counseling, and periodic reassessment of the need for continued elimination should therefore form part of comprehensive food allergy management.

## 6. Future Perspectives

Despite substantial advances in the dietary management of food allergies over the past decade, several important knowledge gaps remain. Future research should prioritize well-designed prospective multicenter studies using standardized diagnostic criteria, harmonized outcome measures, and longer follow-up periods to improve the quality and comparability of the available evidence.

Attention should be directed toward non-IgE-mediated food allergies, where the evidence supporting several dietary management strategies remains largely derived from observational studies and evidence-based recommendations. Robust clinical trials are needed to establish the optimal duration of dietary elimination, define evidence-based food reintroduction protocols, evaluate the long-term nutritional impact of dietary interventions, and identify factors associated with the development of natural tolerance.

Another priority is the identification and validation of reliable biomarkers with the potential to improve diagnosis, monitor disease activity, predict treatment response, and support individualized dietary management. Advances in immunological profiling, metabolomics, genomics, and microbiome research may facilitate earlier diagnosis and more personalized therapeutic strategies.

Emerging approaches, including microbiome-targeted interventions; precision nutrition; and structured food reintroduction strategies such as food ladders, biologic therapies, and combined immunomodulatory treatments, warrant further investigation. Their long-term efficacy, safety, cost-effectiveness, and applicability in routine clinical practice require further evaluation before widespread implementation.

Ultimately, future research should explore increasingly personalized approaches to food allergy management that integrate clinical characteristics, nutritional assessment, molecular biomarkers, and patient-centered outcomes.

## Figures and Tables

**Figure 1:**
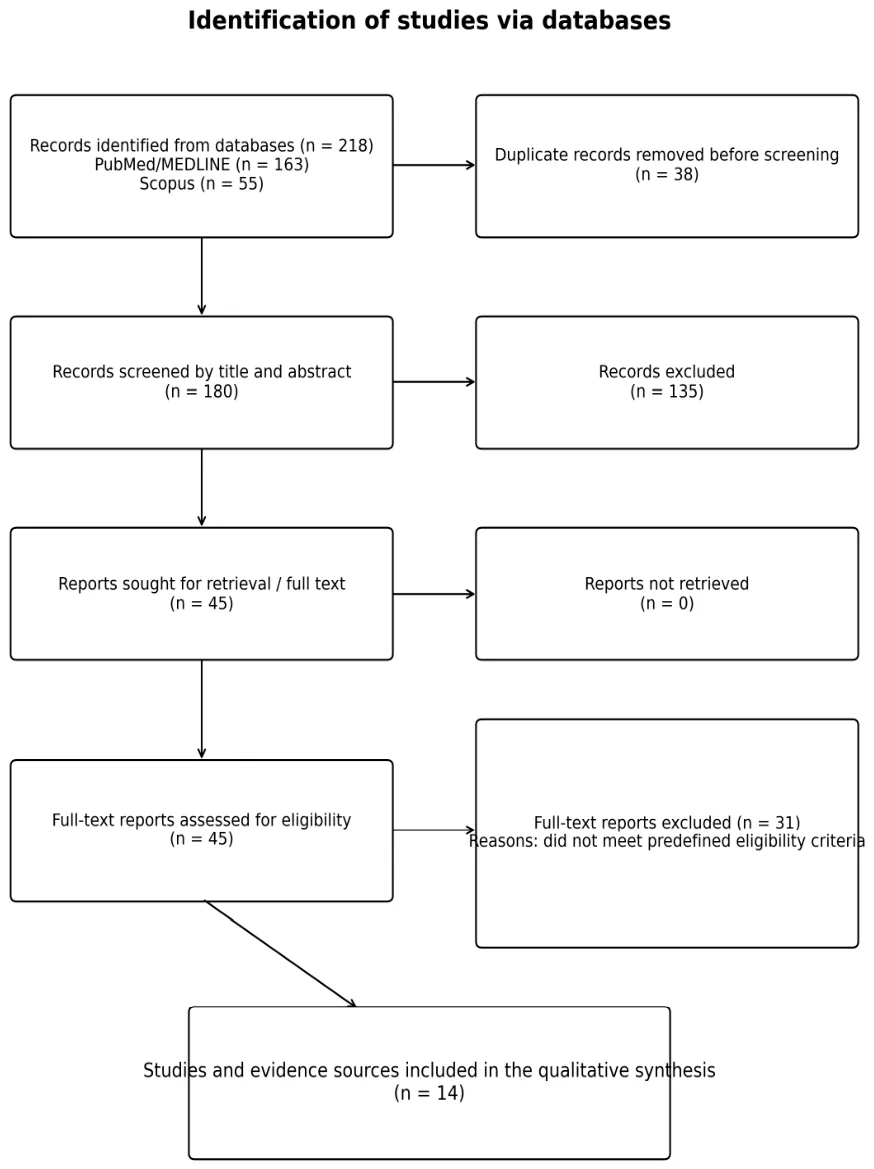
PRISMA 2020 flow diagram of the study selection process.

**Table 1:** Clinical and diagnostic characteristics of IgE-mediated and non-IgE-mediated food allergies.

Characteristic	IgE-Mediated	Non-IgE-Mediated
Immune mechanism	IgE-mediated immune response	Predominantly cell-mediated immune response
Onset	Usually within minutes to 2 h	Usually delayed (hours to days)
Main symptoms	Urticaria, angioedema, gastrointestinal and respiratory symptoms; anaphylaxis may occur	Predominantly gastrointestinal symptoms, depending on the clinical disorder
Diagnostic approach	Clinical history supported by skin-prick testing and/or specific IgE; oral food challenge when required	Clinical history; elimination followed by supervised reintroduction or oral food challenge, depending on the disorder
Treatment	Allergen avoidance; OIT in selected patients	Elimination followed by supervised reintroduction when clinically appropriate

**Table 2:** Characteristics of all studies and evidence sources included in the qualitative synthesis (n = 14).

Study	Study Design	Population	Food Allergy Phenotype	Dietary Intervention/Management Strategy	Main Outcomes	Main Findings
Meyer et al. (2016) [11]	Cohort study	Children with food protein-induced gastrointestinal allergy	Non-IgE-mediated	Elimination diet; nutritional monitoring	Growth and nutrient intake	Elimination diets were associated with nutritional and growth concerns, supporting structured dietetic follow-up.
Scarpone et al. (2023) [12]	Systematic review and meta-analysis	Infants/children in allergenic food introduction studies	Prevention of IgE-mediated food allergy	Timing of allergenic food introduction	Development of IgE-mediated food allergy	Earlier introduction of selected allergenic foods was associated with reduced risk of subsequent IgE-mediated food allergy in relevant populations.
Halken et al. (2021) [17]	EAACI clinical practice guideline	Infants and young children	Prevention of IgE-mediated food allergy	Early allergen introduction	Food allergy prevention	Provides evidence-based recommendations supporting timely introduction of allergenic foods, rather than prolonged avoidance.
Santos et al. (2025) [19]	EAACI clinical practice guideline	Children and adults with IgE-mediated food allergy	IgE-mediated	Allergen avoidance, OIT and comprehensive management	Clinical management and desensitization	Recommends individualized avoidance and active treatment options, including OIT in selected patients.
Meyer et al. (2020) [20]	EAACI position paper	Breastfed infants with non-IgE gastrointestinal food allergy	Non-IgE-mediated	Elimination and supervised reintroduction	Diagnosis, nutritional adequacy and reintroduction	Supports phenotype-specific elimination, nutritional surveillance and reassessment through supervised reintroduction.
Skypala et al. (2023) [27]	EAACI/INDANA position paper and narrative evidence review	Children and adults with food allergy	IgE- and non-IgE-mediated	Individualized dietary management	Nutritional adequacy, dietary diversity and quality of life	Emphasizes dietitian-led individualized care, maintenance of tolerated foods and avoidance of unnecessary restriction.
Riggioni et al. (2024) [28]	Systematic review and meta-analysis	Patients with IgE-mediated food allergy	IgE-mediated	Food allergen immunotherapy/biologic treatment	Efficacy and safety	Immunotherapy improves desensitization outcomes, but requires consideration of treatment-related adverse events.
Vickery et al. (PALISADE) (2018) [29]	Randomized controlled trial	Peanut-allergic children and adolescents	IgE-mediated peanut allergy	AR101 peanut oral immunotherapy	Desensitization/reaction threshold; adverse events	Peanut OIT increased the tolerated dose of peanut compared with placebo, with treatment-related adverse events.
de Silva et al. (2022) [30]	Systematic review and meta-analysis	Patients with IgE-mediated food allergy	IgE-mediated	Allergen immunotherapy and/or biologics	Efficacy and safety	Supports efficacy of active treatment strategies for Desensitization, while highlighting heterogeneity and safety considerations.
Chu et al. (PACE) (2019) [31]	Systematic review and meta-analysis	Patients with peanut allergy	IgE-mediated peanut allergy	Peanut oral immunotherapy	Desensitization and adverse events	Peanut OIT increased desensitization, but also increased treatment-related adverse events compared with avoidance or placebo.
Pajno et al. (2018) [32]	EAACI clinical practice guideline	Patients with IgE-mediated food allergy	IgE-mediated	Allergen immunotherapy	Indications, efficacy and safety	Recommends food allergen immunotherapy only in appropriately selected patients under specialist supervision.
Perkin et al. (EAT) (2016) [33]	Randomized controlled trial	Breastfed infants	Prevention of IgE-mediated food allergy	Early introduction of multiple allergenic foods	Development of food allergy	Early introduction showed benefit, particularly among participants adhering to the intervention protocol.
Soller et al. (2019) [34]	Prospective real-world cohort	Preschool children with peanut allergy	IgE-mediated peanut allergy	Preschool peanut OIT	Safety, feasibility and treatment persistence	Real-world preschool OIT was feasible, but demonstrated the need for careful safety monitoring and patient selection.
Nowak-Wegrzyn et al. (2017) [35]	International consensus guideline	Children with FPIES	Non-IgE-mediated (FPIES)	Elimination and supervised oral food challenge/reintroduction	Diagnosis, acute management and tolerance reassessment	Provides standardized recommendations for avoidance, oral food challenge and reassessment of tolerance in FPIES.

**Table 3:** Summary of dietary interventions in IgE-mediated and non-IgE-mediated food allergies.

Intervention	IgE-Mediated	Non-IgE-Mediated	Benefits	Limitations
Elimination diet	Yes	Yes	Symptom control and prevention of reactions	Risk of nutritional inadequacy with prolonged or multiple-food elimination
Dietitian-led management	Yes	Yes	Improved nutritional adequacy and individualized substitution	Resource intensive; access to specialist dietetic care may vary
Oral immunotherapy	Selected patients	No established role	Desensitization and increased reaction threshold	Treatment-related adverse reactions and treatment burden
Early allergen introduction	Prevention strategy	No established role	Reduced development of selected IgE-mediated food allergies	Requires implementation during infancy; evidence varies by allergen and population
Food/allergen ladder	Selected patients	Selected phenotypes	Gradual food reintroduction and expansion of the diet	Evidence and protocols vary according to the food and clinical phenotype
Supervised food reintroduction after elimination	Yes, when clinically indicated	Yes, when clinically indicated	Reassessment of tolerance and prevention of unnecessary long-term avoidance	Requires individualized assessment and appropriate clinical supervision

## Data Availability

No new datasets were generated or analyzed for this systematic review. All data supporting the findings are derived from the published studies cited in the manuscript and from the review materials provided as Supplementary Files S1 and S2.

## Supplementary Materials

The following supporting information can be downloaded at https://www.mdpi.com/article/10.3390/nu18193180/s1, File S1: Complete database-specific search strategies; File S2: PRISMA 2020 Checklist.
