# Supplementary material for: Dietary Management of IgE-Mediated and Non-IgE-Mediated Food Allergies: A Systematic Review of Current Evidence and Clinical Implications
Source: Nutrients. 2026 Sep 26;18(19):3180. doi: 10.3390/nu18193180 (PMC13666680; doi:10.3390/nu18193180)
Supplement: Supplementary file 1 [file nutrients-18-03180-s001.zip › nutrients-4459787-supplementary.pdf]

Supplementary Materials

Supplementary File S1. Available documented information on the database searches

The electronic searches were performed in PubMed/MEDLINE and Scopus on 10 January 2026 and were restricted by the review eligibility window to studies published from January 2016 through December 2025. The search strategy combined controlled vocabulary, where applicable, and free-text terms addressing food allergy phenotype and dietary management. Principal concepts documented in the review records were: “Food Hypersensitivity”, “Food Allergy”, “IgE-mediated food allergy”, “Non-IgE-mediated food allergy”, “Elimination diet”, “Dietary management”, “Food Protein-Induced Enterocolitis Syndrome (FPIES)”, “Eosinophilic Esophagitis”, “Nutritional management”, “Quality of life”, and “Food allergy quality of life”. Boolean operators AND and OR were used to combine concepts, with syntax adapted to each database. The material below reproduces all search information preserved in the review records and should not be interpreted as a verbatim reconstruction of the original interface-specific query strings.

PubMed/MEDLINE: 163 records were retrieved. The verbatim historical query string, including the exact field tags and any interface-specific syntax used on 10 January 2026, could not be recovered from the archived search records available for this revision. To preserve methodological transparency, it has not been retrospectively reconstructed or presented as an original executed search string.

Scopus: 55 records were retrieved. The verbatim historical query string, including the exact field codes and any interface-specific syntax used on 10 January 2026, could not be recovered from the archived search records available for this revision. To preserve methodological transparency, it has not been retrospectively reconstructed or presented as an original executed search string.

Supplementary File S2. PRISMA 2020 Checklist

Section and Topic	Item	PRISMA 2020 Checklist item	Location where item is reported
TITLE	1	Identify the report as a systematic review.	Title
ABSTRACT	2	See the PRISMA 2020 for Abstracts checklist.	Structured Abstract
INTRODUCTION	3	Describe the rationale for the review in the context of existing knowledge.	Introduction
	4	Provide an explicit statement of the objectives or questions addressed by the review.	End of Introduction (Objectives)
METHODS	5	Specify the inclusion and exclusion criteria	Section 2.3 Eligibility Criteria

		and how studies were grouped.	
	6	Specify all information sources and the date last searched.	Section 2.2 Literature Search Strategy
	7	Present the complete search strategy.	Available documented search information is provided in Supplementary File S1; the unavailable verbatim historical strings and this limitation are reported in Section 2.2 and Supplementary File S1.
	8	Specify the study selection process, reviewers and independence.	Section 2.4 Study Selection
	9	Describe the data collection process.	Section 2.5 Data Extraction
	10a	List and define all outcomes.	Section 2.5 Data Extraction
	10b	List and define all other variables.	Section 2.5 Data Extraction
	11	Specify methods used to assess risk of bias.	Section 2.6 Quality Assessment
	12	Specify effect measures used.	Not applicable (qualitative systematic review without meta-analysis).
	13a	Describe the process used to decide which studies were eligible for each synthesis.	Section 2.7 Data Synthesis
	13b	Describe methods used to prepare data.	Section 2.7 Data Synthesis
	13c	Describe methods used to tabulate or visually display results.	Section 2.7 Data Synthesis; Figures and Tables
	13d	Describe methods used to synthesise results.	Section 2.7 Data Synthesis

	13e	Describe methods used to explore heterogeneity.	Not applicable
	13f	Describe sensitivity analyses.	Not applicable
RESULTS	14	Describe the results of the search and selection process.	Section 3.1 Study Selection; Figure 1 (PRISMA Flow Diagram)
	15	Cite included studies and present their characteristics.	Section 3.2 Characteristics of Included Studies; Table 1
	16a	Present assessments of risk of bias.	Section 3.3 Quality Assessment
	16b	Present results of statistical syntheses.	Not applicable (no meta-analysis).
	16c	Present investigations of heterogeneity.	Not applicable
	16d	Present sensitivity analyses.	Not applicable
	17	Present characteristics of included studies.	Section 3.2; Table 1
	18	Present risk of bias assessments.	Section 3.3 Quality Assessment
	19	Present results for all outcomes.	Sections 3.3–3.9 Results
	20a	Summarise characteristics and risk of bias across studies.	Sections 3.3–3.9 Results
	20b	Present results of all statistical syntheses.	Not applicable
	20c	Present investigations of heterogeneity.	Not applicable
	20d	Present sensitivity analyses.	Not applicable
	21	Present assessments of reporting biases.	Not assessed. Due to the qualitative nature of this review and the absence of meta-analysis, reporting bias was not formally evaluated. This limitation is acknowledged in the Discussion.

	22	Present assessments of certainty of the evidence.	Not assessed. The certainty of evidence (e.g., GRADE) was not formally evaluated because of the heterogeneity of the included studies and the absence of quantitative synthesis. This limitation is discussed in the manuscript.
DISCUSSION	23a	Provide a general interpretation of the results.	Discussion
	23b	Discuss limitations of the evidence.	Strengths and Limitations
	23c	Discuss limitations of the review processes.	Strengths and Limitations
	23d	Discuss implications for practice, policy and future research.	Discussion; Future Directions; Conclusions
OTHER INFORMATION	24a	Provide registration information.	Section 2.1.1 Protocol Registration
	24b	Indicate where the protocol can be accessed.	Not applicable. The review was not prospectively registered and no publicly accessible protocol is available.
	24c	Describe amendments to protocol.	Not applicable
	25	Describe sources of support.	Funding
	26	Declare competing interests.	Conflicts of Interest
	27	Report availability of data, code and materials.	Data Availability Statement
